# Supplementary material for: Respiratory Telerehabilitation of Boys and Young Men with Duchenne Muscular Dystrophy in the COVID-19 Pandemic
Source: Int J Environ Res Public Health. 2021 Jun 8;18(12):6179. doi: 10.3390/ijerph18126179 (PMC8229414; doi:10.3390/ijerph18126179)
Supplement: Supplementary file 1 [file ijerph-18-06179-s001.zip › ijerph-1225541-supplementary.pdf]

Supplementary Table S1. The individual age, VS Score, and BS scale of each of the 45 participants.

Patient	Age	VS	BS
1	13	1	1
2	7	2	1
3	10	3	1
4	12	9	2
5	10	1	1
6	8	2	1
7	17	9	2
8	10	2	2
9	10	2	2
10	16	9	2
11	14	1	1
12	15	9	2
13	24	9	6
14	2,5	2	1
15	4	2	2
16	6	1	2
17	22	9	5
18	7	1	1
19	5,5	1	1
20	8	1	1
21	10	9	1
22	21	9	2
23	24	9	5
24	7	2	2
25	5	1	1
26	7	2	1
27	15	9	3
28	16	4	1
29	4	2	1
30	15	9	3
31	12	9	1
32	2,5	2	1
33	4	2	1
34	14	4	3
35	12	9	2
36	12	3	1
37	12	3	1
38	7	1	1
39	12	3	2
40	4	2	1
41	9	9	1
42	22	9	6
43	14	9	5
44	8	2	1
45	3	2	1
